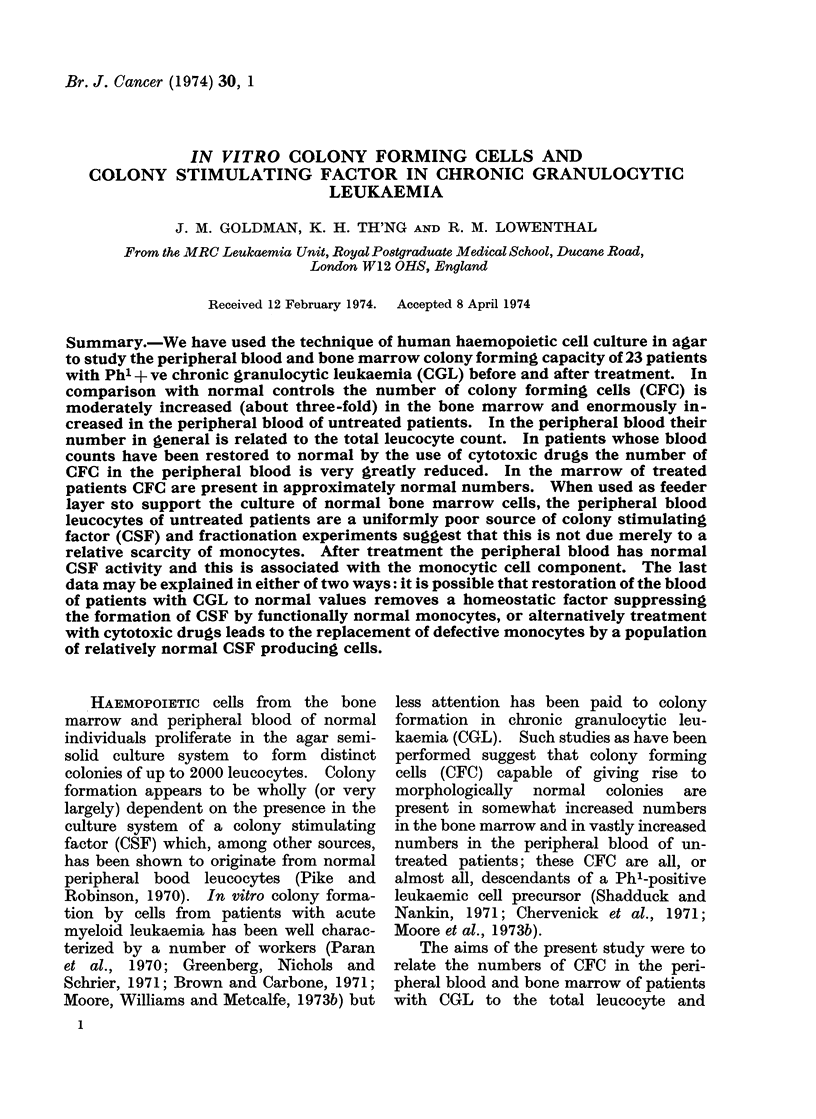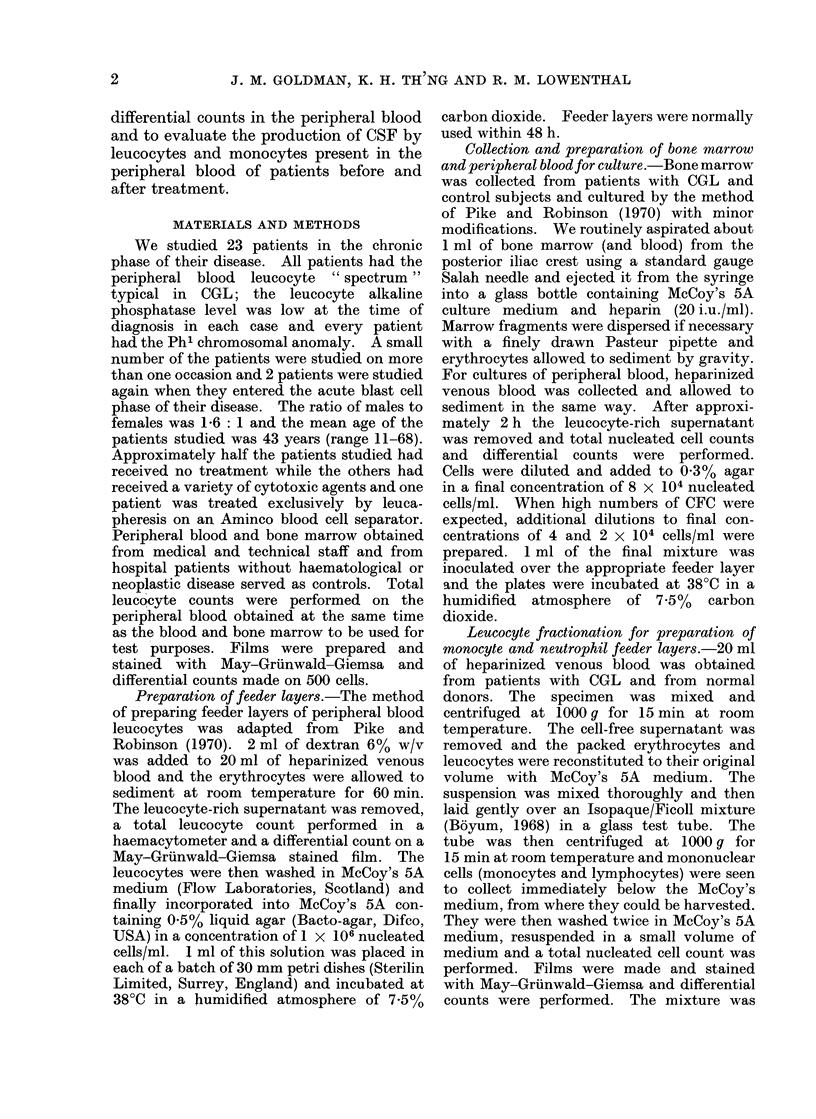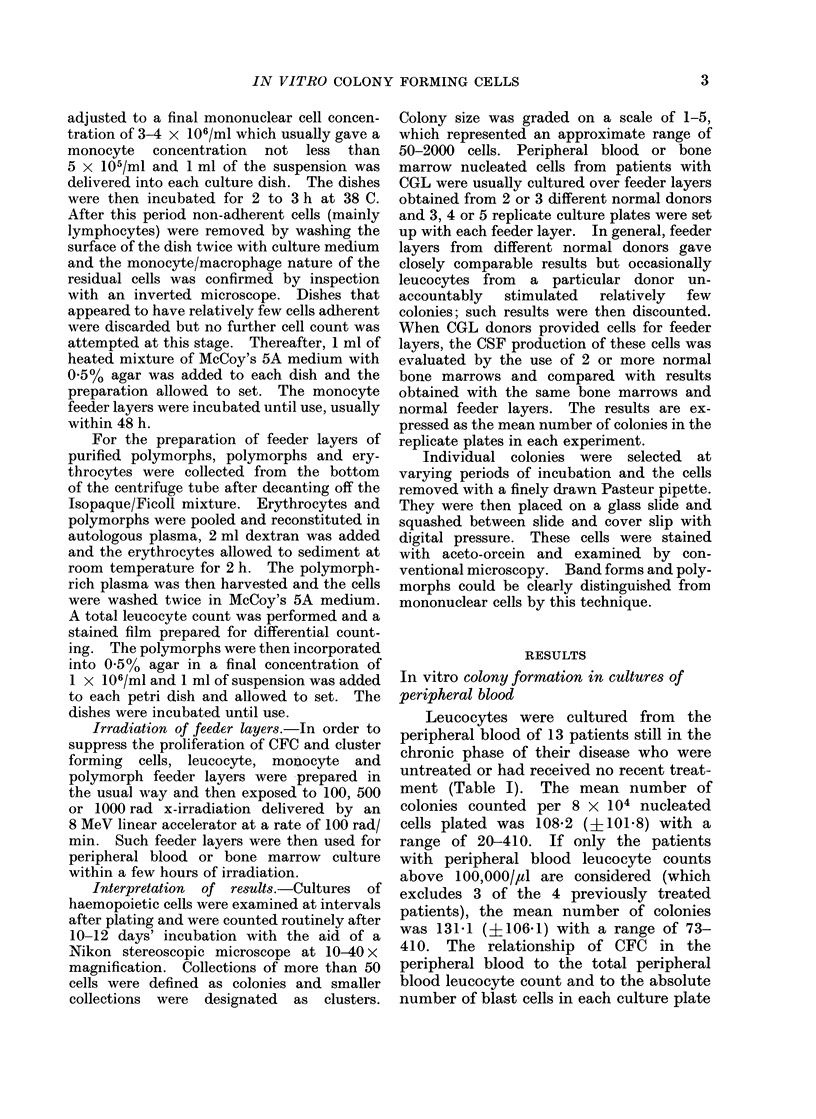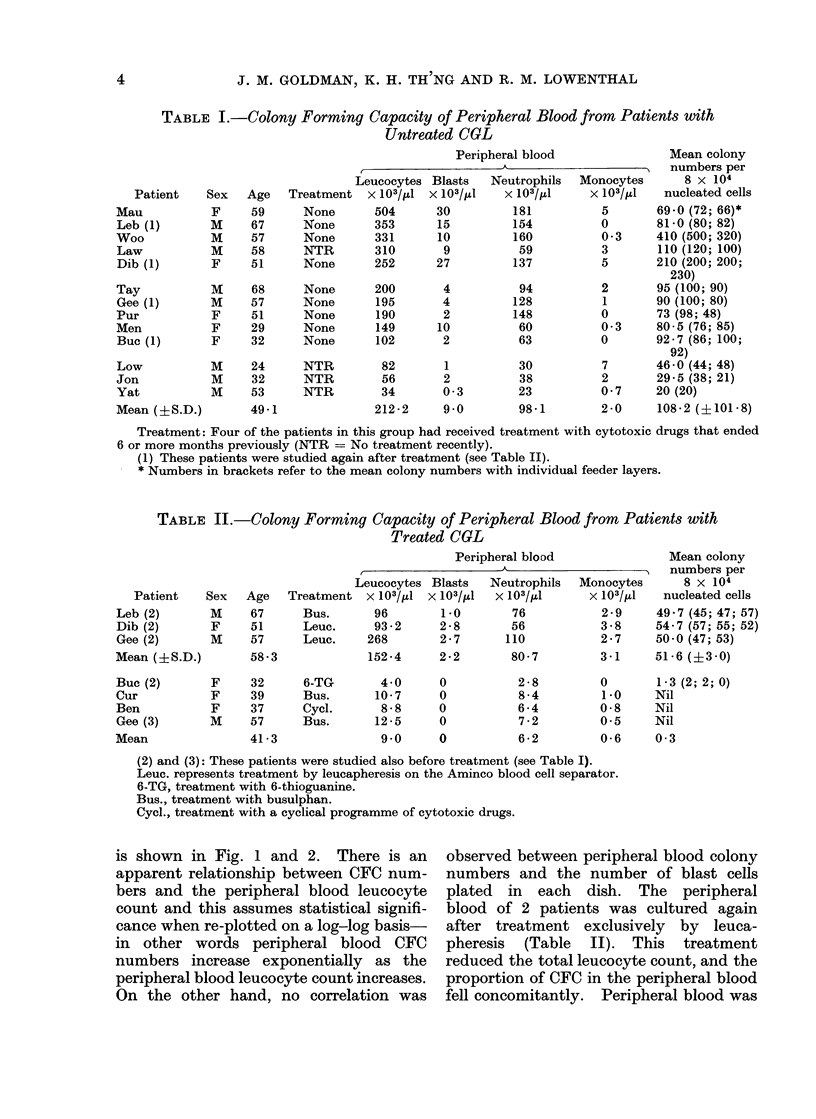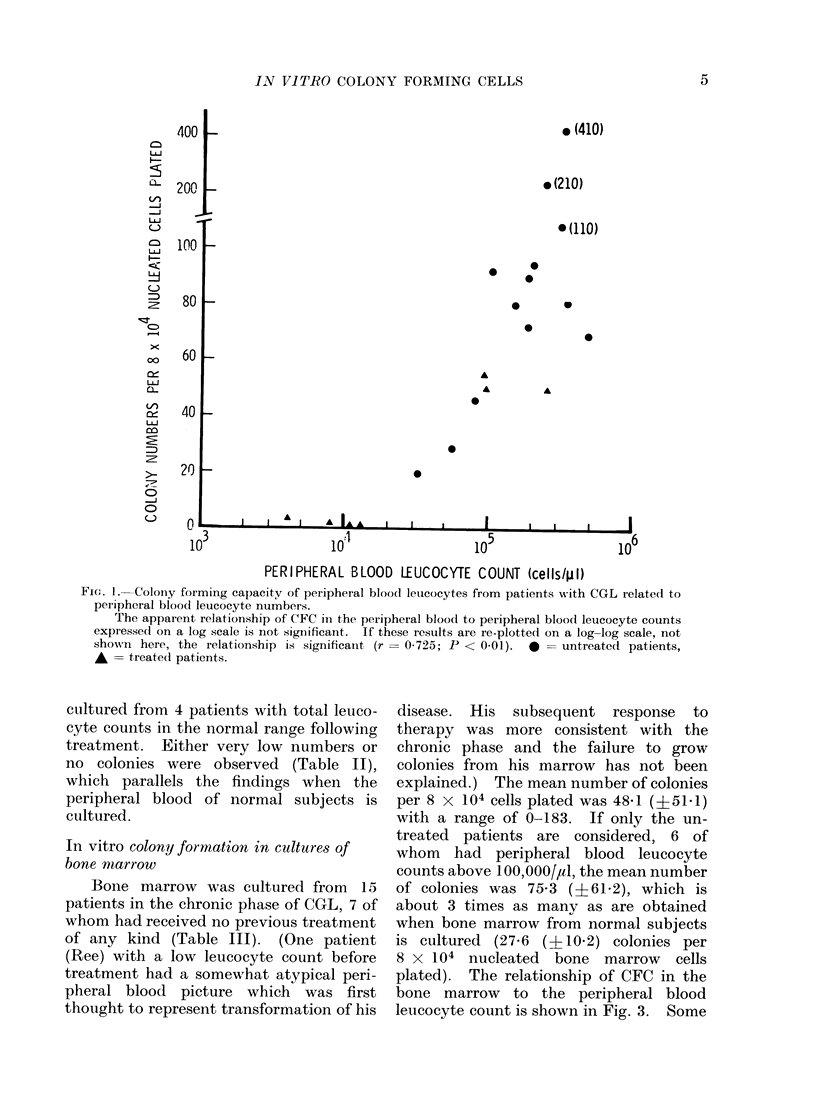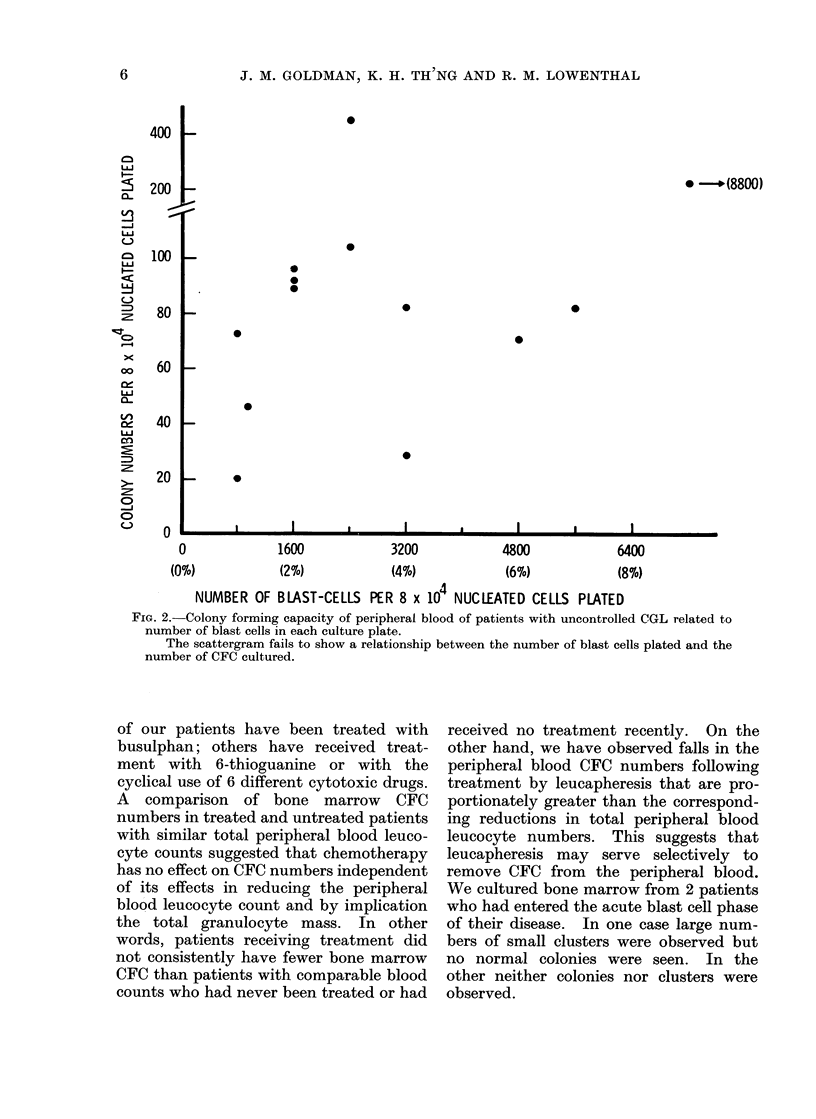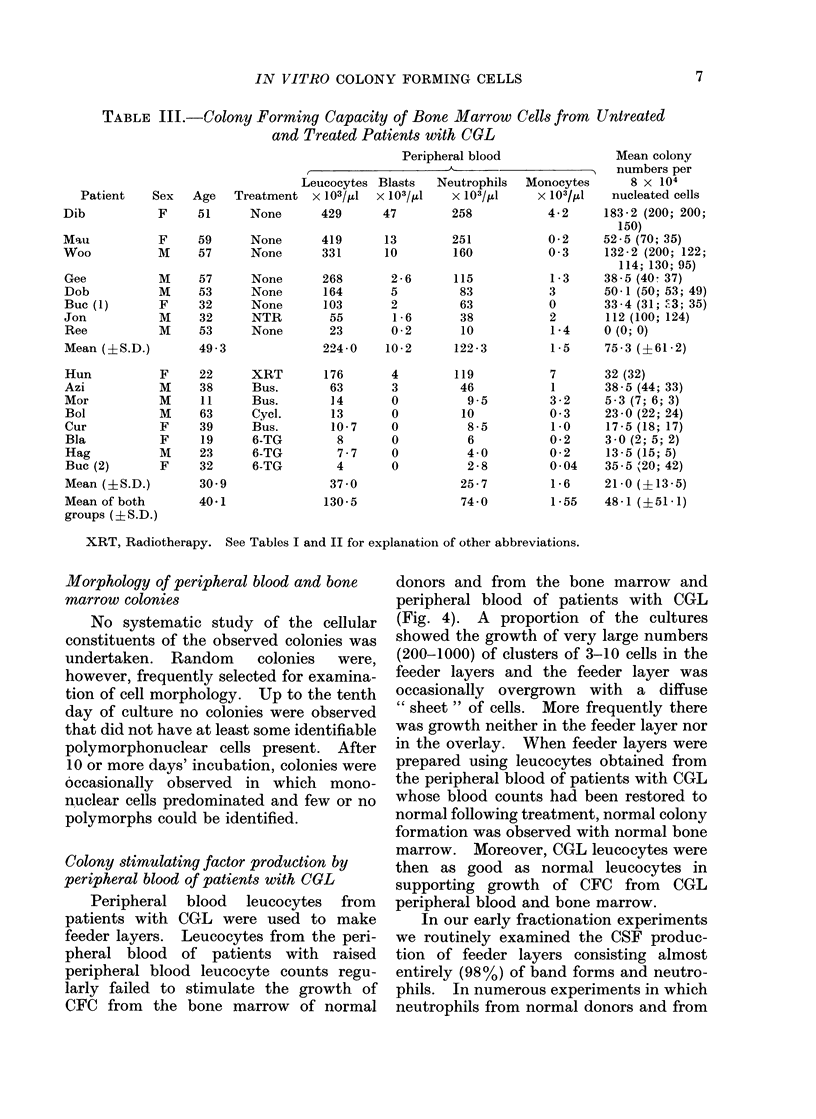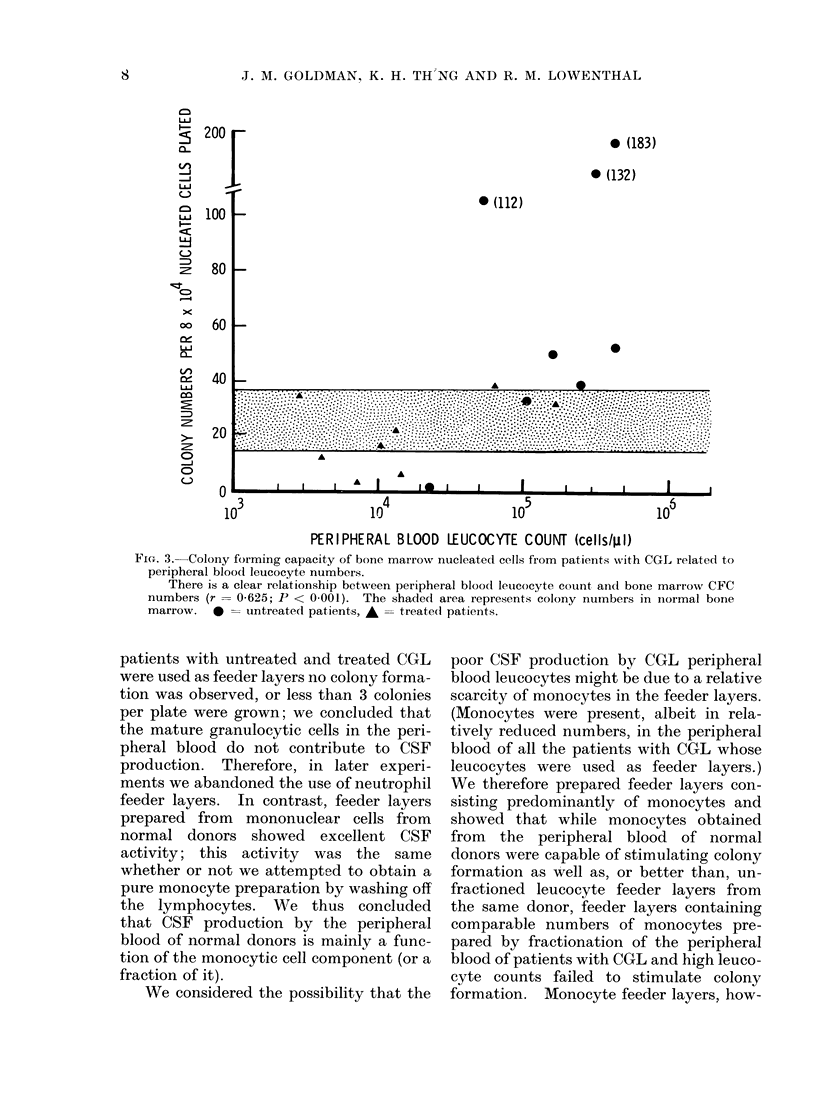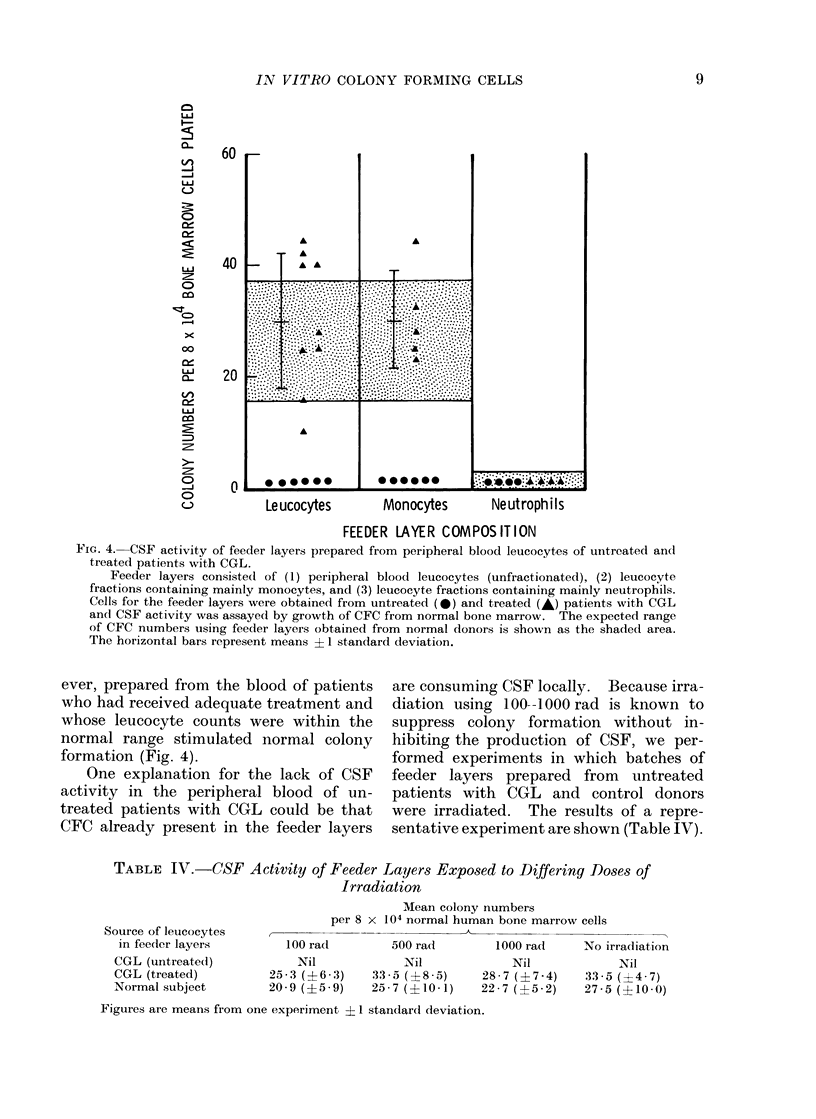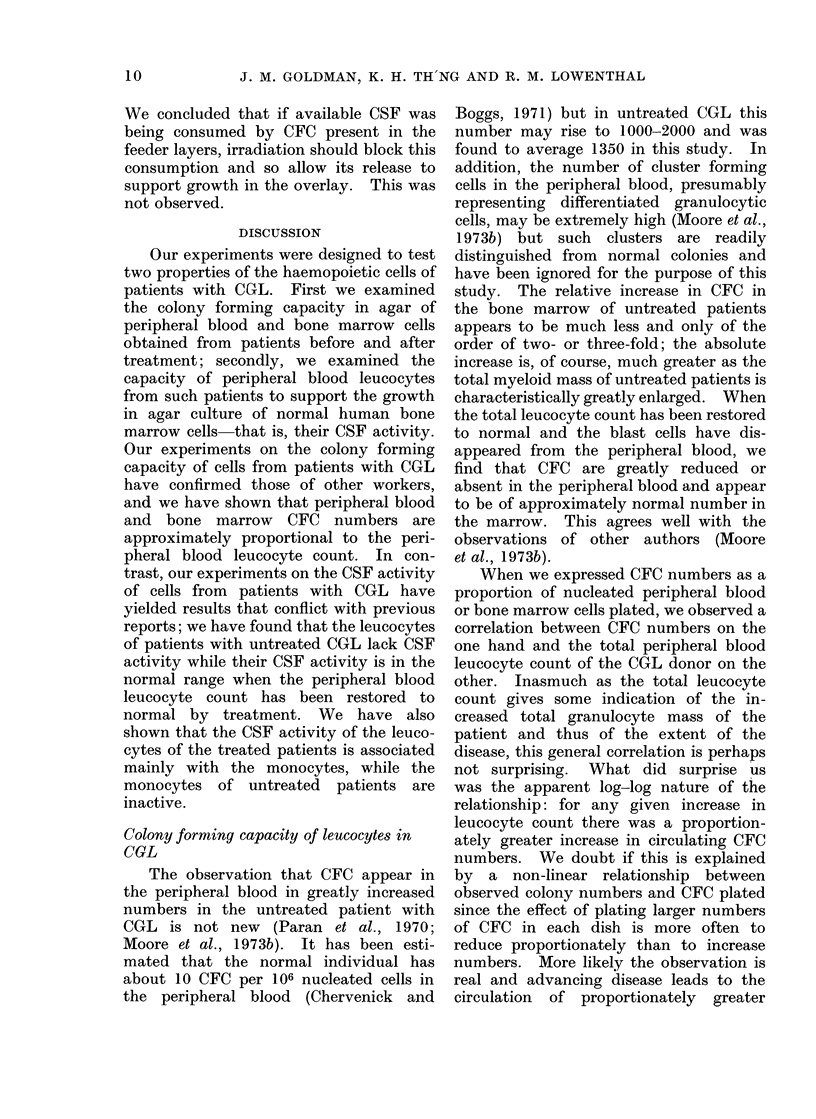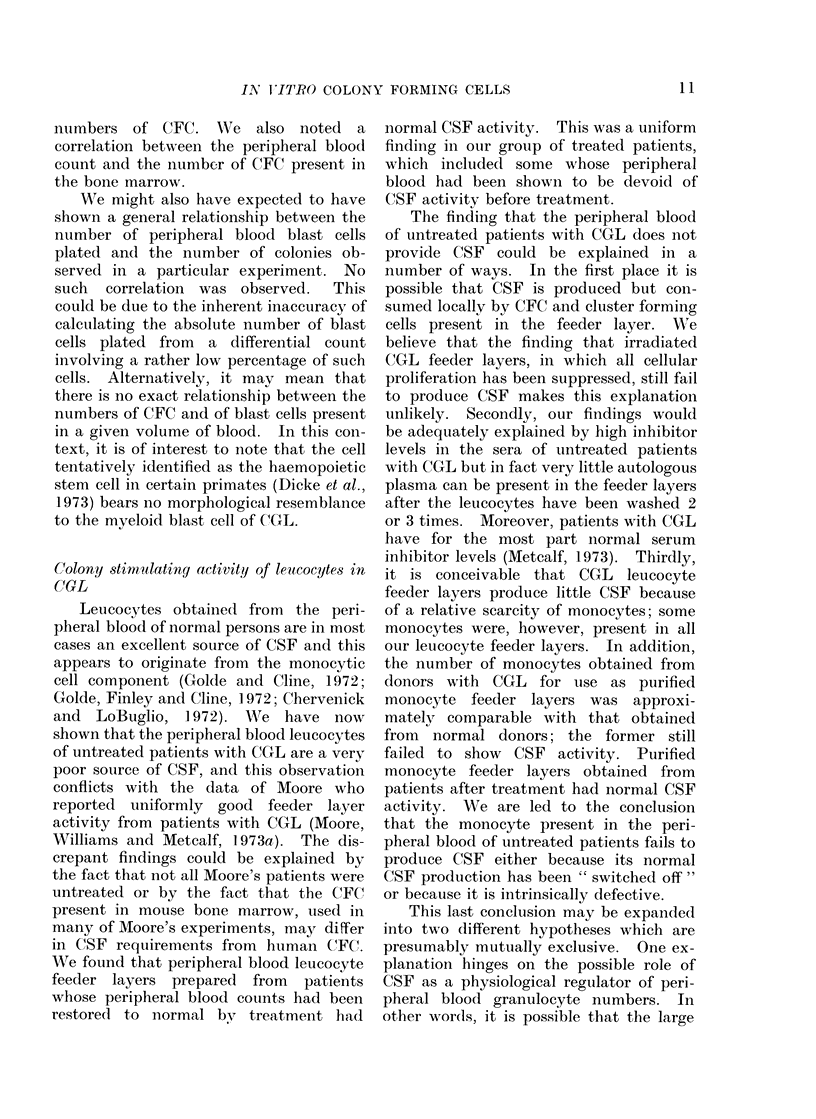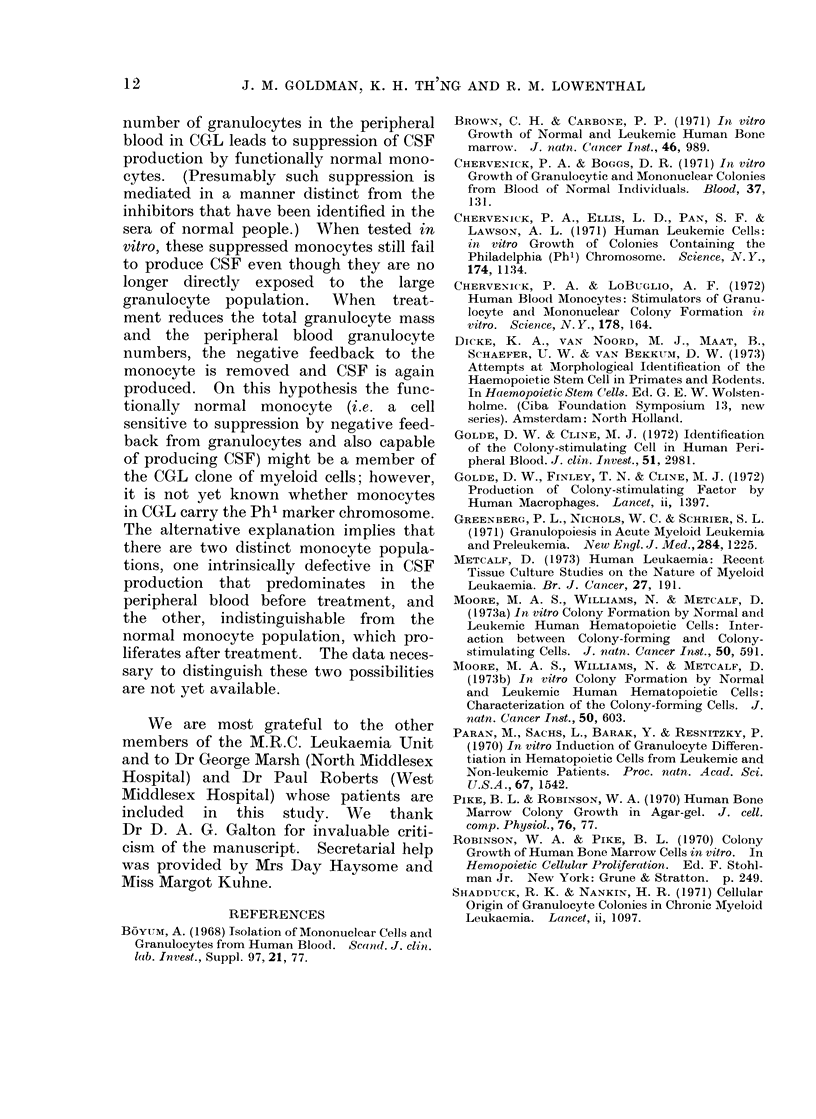# In vitro Colony Forming Cells and Colony Stimulating Factor in Chronic Granulocytic Leukaemia

**DOI:** 10.1038/bjc.1974.108

**Published:** 1974-07

**Authors:** J. M. Goldman, K. H. Th'ng, R. M. Lowenthal

## Abstract

We have used the technique of human haemopoietic cell culture in agar to study the peripheral blood and bone marrow colony forming capacity of 23 patients with Ph^1^ + ve chronic granulocytic leukaemia (CGL) before and after treatment. In comparison with normal controls the number of colony forming cells (CFC) is moderately increased (about three-fold) in the bone marrow and enormously increased in the peripheral blood of untreated patients. In the peripheral blood their number in general is related to the total leucocyte count. In patients whose blood counts have been restored to normal by the use of cytotoxic drugs the number of CFC in the peripheral blood is very greatly reduced. In the marrow of treated patients CFC are present in approximately normal numbers. When used as feeder layer sto support the culture of normal bone marrow cells, the peripheral blood leucocytes of untreated patients are a uniformly poor source of colony stimulating factor (CSF) and fractionation experiments suggest that this is not due merely to a relative scarcity of monocytes. After treatment the peripheral blood has normal CSF activity and this is associated with the monocytic cell component. The last data may be explained in either of two ways: it is possible that restoration of the blood of patients with CGL to normal values removes a homeostatic factor suppressing the formation of CSF by functionally normal monocytes, or alternatively treatment with cytotoxic drugs leads to the replacement of defective monocytes by a population of relatively normal CSF producing cells.


*Br. J. Cancer* (1974) **30**, 1

### *IN VITRO* COLONY FORMING CELLS AND COLONY STIMULATING FACTOR IN CHRONIC GRANULOCYTIC LEUKAEMIA

J. M. GOLDMAN, K. H. TH'NG AND R. M. LOWENTHAL

*From the MRC Leukaemia Unit, Royal Postgraduate Medical School, Ducane Road, London W12 OHS, England*

Received 12 February 1974. Accepted 8 April 1974

**Summary.—We have used the technique of human haemopoietic cell culture in agar to study the peripheral blood and bone marrow colony forming capacity of 23 patients with Ph[1] + ve chronic granulocytic leukaemia (CGL) before and after treatment. In comparison with normal controls the number of colony forming cells (CFC) is moderately increased (about three-fold) in the bone marrow and enormously increased in the peripheral blood of untreated patients. In the peripheral blood their number in general is related to the total leucocyte count. In patients whose blood counts have been restored to normal by the use of cytotoxic drugs the number of CFC in the peripheral blood is very greatly reduced. In the marrow of treated patients CFC are present in approximately normal numbers. When used as feeder layer sto support the culture of normal bone marrow cells, the peripheral blood leucocytes of untreated patients are a uniformly poor source of colony stimulating factor (CSF) and fractionation experiments suggest that this is not due merely to a relative scarcity of monocytes. After treatment the peripheral blood has normal CSF activity and this is associated with the monocytic cell component. The last data may be explained in either of two ways: it is possible that restoration of the blood of patients with CGL to normal values removes a homeostatic factor suppressing the formation of CSF by functionally normal monocytes, or alternatively treatment with cytotoxic drugs leads to the replacement of defective monocytes by a population of relatively normal CSF producing cells.**

HAEMOPOIETIC cells from the bone marrow and peripheral blood of normal individuals proliferate in the agar semi-solid culture system to form distinct colonies of up to 2000 leucocytes. Colony formation appears to be wholly (or very largely) dependent on the presence in the culture system of a colony stimulating factor (CSF) which, among other sources, has been shown to originate from normal peripheral bood leucocytes (Pike and Robinson, 1970). *In vitro* colony formation by cells from patients with acute myeloid leukaemia has been well characterized by a number of workers (Paran *et al.*, 1970; Greenberg, Nichols and Schrier, 1971; Brown and Carbone, 1971; Moore, Williams and Metcalfe, 1973*b*) but less attention has been paid to colony formation in chronic granulocytic leukaemia (CGL). Such studies as have been performed suggest that colony forming cells (CFC) capable of giving rise to morphologically normal colonies are present in somewhat increased numbers in the bone marrow and in vastly increased numbers in the peripheral blood of untreated patients; these CFC are all, or almost all, descendants of a Ph[1]-positive leukaemic cell precursor (Shadduck and Nankin, 1971; Chervenick *et al.*, 1971; Moore *et al.*, 1973*b*).

The aims of the present study were to relate the numbers of CFC in the peripheral blood and bone marrow of patients with CGL to the total leucocyte and differential counts in the peripheral blood and to evaluate the production of CSF by leucocytes and monocytes present in the peripheral blood of patients before and after treatment.

### MATERIALS AND METHODS

We studied 23 patients in the chronic phase of their disease. All patients had the peripheral blood leucocyte "spectrum" typical in CGL; the leucocyte alkaline phosphatase level was low at the time of diagnosis in each case and every patient had the $Ph^1$ chromosomal anomaly. A small number of the patients were studied on more than one occasion and 2 patients were studied again when they entered the acute blast cell phase of their disease. The ratio of males to females was 1·6 : 1 and the mean age of the patients studied was 43 years (range 11–68). Approximately half the patients studied had received no treatment while the others had received a variety of cytotoxic agents and one patient was treated exclusively by leucapheresis on an Aminco blood cell separator. Peripheral blood and bone marrow obtained from medical and technical staff and from hospital patients without haematological or neoplastic disease served as controls. Total leucocyte counts were performed on the peripheral blood obtained at the same time as the blood and bone marrow to be used for test purposes. Films were prepared and stained with May–Grünwald–Giemsa and differential counts made on 500 cells.

*Preparation of feeder layers.*—The method of preparing feeder layers of peripheral blood leucocytes was adapted from Pike and Robinson (1970). 2 ml of dextran 6% w/v was added to 20 ml of heparinized venous blood and the erythrocytes were allowed to sediment at room temperature for 60 min. The leucocyte-rich supernatant was removed, a total leucocyte count performed in a haemacytometer and a differential count on a May–Grünwald–Giemsa stained film. The leucocytes were then washed in McCoy's 5A medium (Flow Laboratories, Scotland) and finally incorporated into McCoy's 5A containing 0·5% liquid agar (Bacto-agar, Difco, USA) in a concentration of $1 \times 10^6$ nucleated cells/ml. 1 ml of this solution was placed in each of a batch of 30 mm petri dishes (Sterilin Limited, Surrey, England) and incubated at 38°C in a humidified atmosphere of 7·5% carbon dioxide. Feeder layers were normally used within 48 h.

*Collection and preparation of bone marrow and peripheral blood for culture.*—Bone marrow was collected from patients with CGL and control subjects and cultured by the method of Pike and Robinson (1970) with minor modifications. We routinely aspirated about 1 ml of bone marrow (and blood) from the posterior iliac crest using a standard gauge Salah needle and ejected it from the syringe into a glass bottle containing McCoy's 5A culture medium and heparin (20 i.u./ml). Marrow fragments were dispersed if necessary with a finely drawn Pasteur pipette and erythrocytes allowed to sediment by gravity. For cultures of peripheral blood, heparinized venous blood was collected and allowed to sediment in the same way. After approximately 2 h the leucocyte-rich supernatant was removed and total nucleated cell counts and differential counts were performed. Cells were diluted and added to 0·3% agar in a final concentration of $8 \times 10^4$ nucleated cells/ml. When high numbers of CFC were expected, additional dilutions to final concentrations of 4 and $2 \times 10^4$ cells/ml were prepared. 1 ml of the final mixture was inoculated over the appropriate feeder layer and the plates were incubated at 38°C in a humidified atmosphere of 7·5% carbon dioxide.

*Leucocyte fractionation for preparation of monocyte and neutrophil feeder layers.*—20 ml of heparinized venous blood was obtained from patients with CGL and from normal donors. The specimen was mixed and centrifuged at 1000 *g* for 15 min at room temperature. The cell-free supernatant was removed and the packed erythrocytes and leucocytes were reconstituted to their original volume with McCoy's 5A medium. The suspension was mixed thoroughly and then laid gently over an Isopaque/Ficoll mixture (Böyum, 1968) in a glass test tube. The tube was then centrifuged at 1000 *g* for 15 min at room temperature and mononuclear cells (monocytes and lymphocytes) were seen to collect immediately below the McCoy's medium, from where they could be harvested. They were then washed twice in McCoy's 5A medium, resuspended in a small volume of medium and a total nucleated cell count was performed. Films were made and stained with May–Grünwald–Giemsa and differential counts were performed. The mixture was adjusted to a final mononuclear cell concentration of 3–4 $\times$ $10^6$/ml which usually gave a monocyte concentration not less than 5 $\times$ $10^5$/ml and 1 ml of the suspension was delivered into each culture dish. The dishes were then incubated for 2 to 3 h at 38 C. After this period non-adherent cells (mainly lymphocytes) were removed by washing the surface of the dish twice with culture medium and the monocyte/macrophage nature of the residual cells was confirmed by inspection with an inverted microscope. Dishes that appeared to have relatively few cells adherent were discarded but no further cell count was attempted at this stage. Thereafter, 1 ml of heated mixture of McCoy's 5A medium with 0·5% agar was added to each dish and the preparation allowed to set. The monocyte feeder layers were incubated until use, usually within 48 h.

For the preparation of feeder layers of purified polymorphs, polymorphs and erythrocytes were collected from the bottom of the centrifuge tube after decanting off the Isopaque/Ficoll mixture. Erythrocytes and polymorphs were pooled and reconstituted in autologous plasma, 2 ml dextran was added and the erythrocytes allowed to sediment at room temperature for 2 h. The polymorph-rich plasma was then harvested and the cells were washed twice in McCoy's 5A medium. A total leucocyte count was performed and a stained film prepared for differential counting. The polymorphs were then incorporated into 0·5% agar in a final concentration of 1 $\times$ $10^6$/ml and 1 ml of suspension was added to each petri dish and allowed to set. The dishes were incubated until use.

*Irradiation of feeder layers.*—In order to suppress the proliferation of CFC and cluster forming cells, leucocyte, monocyte and polymorph feeder layers were prepared in the usual way and then exposed to 100, 500 or 1000 rad x-irradiation delivered by an 8 MeV linear accelerator at a rate of 100 rad/min. Such feeder layers were then used for peripheral blood or bone marrow culture within a few hours of irradiation.

*Interpretation of results.*—Cultures of haemopoietic cells were examined at intervals after plating and were counted routinely after 10–12 days' incubation with the aid of a Nikon stereoscopic microscope at 10–40$\times$ magnification. Collections of more than 50 cells were defined as colonies and smaller collections were designated as clusters. Colony size was graded on a scale of 1–5, which represented an approximate range of 50–2000 cells. Peripheral blood or bone marrow nucleated cells from patients with CGL were usually cultured over feeder layers obtained from 2 or 3 different normal donors and 3, 4 or 5 replicate culture plates were set up with each feeder layer. In general, feeder layers from different normal donors gave closely comparable results but occasionally leucocytes from a particular donor unaccountably stimulated relatively few colonies; such results were then discounted. When CGL donors provided cells for feeder layers, the CSF production of these cells was evaluated by the use of 2 or more normal bone marrows and compared with results obtained with the same bone marrows and normal feeder layers. The results are expressed as the mean number of colonies in the replicate plates in each experiment.

Individual colonies were selected at varying periods of incubation and the cells removed with a finely drawn Pasteur pipette. They were then placed on a glass slide and squashed between slide and cover slip with digital pressure. These cells were stained with aceto-orcein and examined by conventional microscopy. Band forms and polymorphs could be clearly distinguished from mononuclear cells by this technique.

### RESULTS

### In vitro *colony formation in cultures of peripheral blood*

Leucocytes were cultured from the peripheral blood of 13 patients still in the chronic phase of their disease who were untreated or had received no recent treatment (Table I). The mean number of colonies counted per 8 $\times$ $10^4$ nucleated cells plated was 108·2 ($\pm$101·8) with a range of 20–410. If only the patients with peripheral blood leucocyte counts above 100,000/$\mu$l are considered (which excludes 3 of the 4 previously treated patients), the mean number of colonies was 131·1 ($\pm$106·1) with a range of 73–410. The relationship of CFC in the peripheral blood to the total peripheral blood leucocyte count and to the absolute number of blast cells in each culture plate

TABLE I.—*Colony Forming Capacity of Peripheral Blood from Patients with Untreated CGL*

| Patient | Sex | Age | Treatment | Peripheral blood: Leucocytes $\times 10^3/\mu l$ | Blasts $\times 10^3/\mu l$ | Neutrophils $\times 10^3/\mu l$ | Monocytes $\times 10^3/\mu l$ | Mean colony numbers per $8 \times 10^4$ nucleated cells |
|---|---|---|---|---|---|---|---|---|
| Mau | F | 59 | None | 504 | 30 | 181 | 5 | 69·0 (72; 66)* |
| Leb (1) | M | 67 | None | 353 | 15 | 154 | 0 | 81·0 (80; 82) |
| Woo | M | 57 | None | 331 | 10 | 160 | 0·3 | 410 (500; 320) |
| Law | M | 58 | NTR | 310 | 9 | 59 | 3 | 110 (120; 100) |
| Dib (1) | F | 51 | None | 252 | 27 | 137 | 5 | 210 (200; 200; 230) |
| Tay | M | 68 | None | 200 | 4 | 94 | 2 | 95 (100; 90) |
| Gee (1) | M | 57 | None | 195 | 4 | 128 | 1 | 90 (100; 80) |
| Pur | F | 51 | None | 190 | 2 | 148 | 0 | 73 (98; 48) |
| Men | F | 29 | None | 149 | 10 | 60 | 0·3 | 80·5 (76; 85) |
| Buc (1) | F | 32 | None | 102 | 2 | 63 | 0 | 92·7 (86; 100; 92) |
| Low | M | 24 | NTR | 82 | 1 | 30 | 7 | 46·0 (44; 48) |
| Jon | M | 32 | NTR | 56 | 2 | 38 | 2 | 29·5 (38; 21) |
| Yat | M | 53 | NTR | 34 | 0·3 | 23 | 0·7 | 20 (20) |
| Mean (±S.D.) | | 49·1 | | 212·2 | 9·0 | 98·1 | 2·0 | 108·2 (±101·8) |

Treatment: Four of the patients in this group had received treatment with cytotoxic drugs that ended 6 or more months previously (NTR = No treatment recently).

(1) These patients were studied again after treatment (see Table II).

* Numbers in brackets refer to the mean colony numbers with individual feeder layers.

TABLE II.—*Colony Forming Capacity of Peripheral Blood from Patients with Treated CGL*

| Patient | Sex | Age | Treatment | Peripheral blood: Leucocytes $\times 10^3/\mu l$ | Blasts $\times 10^3/\mu l$ | Neutrophils $\times 10^3/\mu l$ | Monocytes $\times 10^3/\mu l$ | Mean colony numbers per $8 \times 10^4$ nucleated cells |
|---|---|---|---|---|---|---|---|---|
| Leb (2) | M | 67 | Bus. | 96 | 1·0 | 76 | 2·9 | 49·7 (45; 47; 57) |
| Dib (2) | F | 51 | Leuc. | 93·2 | 2·8 | 56 | 3·8 | 54·7 (57; 55; 52) |
| Gee (2) | M | 57 | Leuc. | 268 | 2·7 | 110 | 2·7 | 50·0 (47; 53) |
| Mean (±S.D.) | | 58·3 | | 152·4 | 2·2 | 80·7 | 3·1 | 51·6 (±3·0) |
| Buc (2) | F | 32 | 6-TG | 4·0 | 0 | 2·8 | 0 | 1·3 (2; 2; 0) |
| Cur | F | 39 | Bus. | 10·7 | 0 | 8·4 | 1·0 | Nil |
| Ben | F | 37 | Cycl. | 8·8 | 0 | 6·4 | 0·8 | Nil |
| Gee (3) | M | 57 | Bus. | 12·5 | 0 | 7·2 | 0·5 | Nil |
| Mean | | 41·3 | | 9·0 | 0 | 6·2 | 0·6 | 0·3 |

(2) and (3): These patients were studied also before treatment (see Table I).

Leuc. represents treatment by leucapheresis on the Aminco blood cell separator.

6-TG, treatment with 6-thioguanine.

Bus., treatment with busulphan.

Cycl., treatment with a cyclical programme of cytotoxic drugs.

is shown in Fig. 1 and 2. There is an apparent relationship between CFC numbers and the peripheral blood leucocyte count and this assumes statistical significance when re-plotted on a log–log basis—in other words peripheral blood CFC numbers increase exponentially as the peripheral blood leucocyte count increases. On the other hand, no correlation was observed between peripheral blood colony numbers and the number of blast cells plated in each dish. The peripheral blood of 2 patients was cultured again after treatment exclusively by leucapheresis (Table II). This treatment reduced the total leucocyte count, and the proportion of CFC in the peripheral blood fell concomitantly. Peripheral blood was

FIG. 1.—Colony forming capacity of peripheral blood leucocytes from patients with CGL related to peripheral blood leucocyte numbers.

The apparent relationship of CFC in the peripheral blood to peripheral blood leucocyte counts expressed on a log scale is not significant. If these results are re-plotted on a log–log scale, not shown here, the relationship is significant ($r = 0{\cdot}725$; $P < 0{\cdot}01$). ● = untreated patients, ▲ = treated patients.

cultured from 4 patients with total leucocyte counts in the normal range following treatment. Either very low numbers or no colonies were observed (Table II), which parallels the findings when the peripheral blood of normal subjects is cultured.

### In vitro *colony formation in cultures of bone marrow*

Bone marrow was cultured from 15 patients in the chronic phase of CGL, 7 of whom had received no previous treatment of any kind (Table III). (One patient (Ree) with a low leucocyte count before treatment had a somewhat atypical peripheral blood picture which was first thought to represent transformation of his disease. His subsequent response to therapy was more consistent with the chronic phase and the failure to grow colonies from his marrow has not been explained.) The mean number of colonies per $8 \times 10^4$ cells plated was 48·1 ($\pm$51·1) with a range of 0–183. If only the untreated patients are considered, 6 of whom had peripheral blood leucocyte counts above 100,000/$\mu$l, the mean number of colonies was 75·3 ($\pm$61·2), which is about 3 times as many as are obtained when bone marrow from normal subjects is cultured (27·6 ($\pm$10·2) colonies per $8 \times 10^4$ nucleated bone marrow cells plated). The relationship of CFC in the bone marrow to the peripheral blood leucocyte count is shown in Fig. 3. Some

FIG. 2.—Colony forming capacity of peripheral blood of patients with uncontrolled CGL related to number of blast cells in each culture plate.

The scattergram fails to show a relationship between the number of blast cells plated and the number of CFC cultured.

of our patients have been treated with busulphan; others have received treatment with 6-thioguanine or with the cyclical use of 6 different cytotoxic drugs. A comparison of bone marrow CFC numbers in treated and untreated patients with similar total peripheral blood leucocyte counts suggested that chemotherapy has no effect on CFC numbers independent of its effects in reducing the peripheral blood leucocyte count and by implication the total granulocyte mass. In other words, patients receiving treatment did not consistently have fewer bone marrow CFC than patients with comparable blood counts who had never been treated or had received no treatment recently. On the other hand, we have observed falls in the peripheral blood CFC numbers following treatment by leucapheresis that are proportionately greater than the corresponding reductions in total peripheral blood leucocyte numbers. This suggests that leucapheresis may serve selectively to remove CFC from the peripheral blood. We cultured bone marrow from 2 patients who had entered the acute blast cell phase of their disease. In one case large numbers of small clusters were observed but no normal colonies were seen. In the other neither colonies nor clusters were observed.

TABLE III.—*Colony Forming Capacity of Bone Marrow Cells from Untreated and Treated Patients with CGL*

| Patient | Sex | Age | Treatment | Peripheral blood: Leucocytes $\times 10^3/\mu l$ | Blasts $\times 10^3/\mu l$ | Neutrophils $\times 10^3/\mu l$ | Monocytes $\times 10^3/\mu l$ | Mean colony numbers per $8 \times 10^4$ nucleated cells |
|---|---|---|---|---|---|---|---|---|
| Dib | F | 51 | None | 429 | 47 | 258 | 4·2 | 183·2 (200; 200; 150) |
| Mau | F | 59 | None | 419 | 13 | 251 | 0·2 | 52·5 (70; 35) |
| Woo | M | 57 | None | 331 | 10 | 160 | 0·3 | 132·2 (200; 122; 114; 130; 95) |
| Gee | M | 57 | None | 268 | 2·6 | 115 | 1·3 | 38·5 (40: 37) |
| Dob | M | 53 | None | 164 | 5 | 83 | 3 | 50·1 (50; 53; 49) |
| Buc (1) | F | 32 | None | 103 | 2 | 63 | 0 | 33·4 (31; 33; 35) |
| Jon | M | 32 | NTR | 55 | 1·6 | 38 | 2 | 112 (100; 124) |
| Ree | M | 53 | None | 23 | 0·2 | 10 | 1·4 | 0 (0; 0) |
| Mean (±S.D.) | | 49·3 | | 224·0 | 10·2 | 122·3 | 1·5 | 75·3 (±61·2) |
| Hun | F | 22 | XRT | 176 | 4 | 119 | 7 | 32 (32) |
| Azi | M | 38 | Bus. | 63 | 3 | 46 | 1 | 38·5 (44; 33) |
| Mor | M | 11 | Bus. | 14 | 0 | 9·5 | 3·2 | 5·3 (7; 6; 3) |
| Bol | M | 63 | Cycl. | 13 | 0 | 10 | 0·3 | 23·0 (22; 24) |
| Cur | F | 39 | Bus. | 10·7 | 0 | 8·5 | 1·0 | 17·5 (18; 17) |
| Bla | F | 19 | 6-TG | 8 | 0 | 6 | 0·2 | 3·0 (2; 5; 2) |
| Hag | M | 23 | 6-TG | 7·7 | 0 | 4·0 | 0·2 | 13·5 (15; 5) |
| Buc (2) | F | 32 | 6-TG | 4 | 0 | 2·8 | 0·04 | 35·5 (20; 42) |
| Mean (±S.D.) | | 30·9 | | 37·0 | | 25·7 | 1·6 | 21·0 (±13·5) |
| Mean of both groups (±S.D.) | | 40·1 | | 130·5 | | 74·0 | 1·55 | 48·1 (±51·1) |

XRT, Radiotherapy. See Tables I and II for explanation of other abbreviations.

*Morphology of peripheral blood and bone marrow colonies*

No systematic study of the cellular constituents of the observed colonies was undertaken. Random colonies were, however, frequently selected for examination of cell morphology. Up to the tenth day of culture no colonies were observed that did not have at least some identifiable polymorphonuclear cells present. After 10 or more days' incubation, colonies were occasionally observed in which mononuclear cells predominated and few or no polymorphs could be identified.

*Colony stimulating factor production by peripheral blood of patients with CGL*

Peripheral blood leucocytes from patients with CGL were used to make feeder layers. Leucocytes from the peripheral blood of patients with raised peripheral blood leucocyte counts regularly failed to stimulate the growth of CFC from the bone marrow of normal donors and from the bone marrow and peripheral blood of patients with CGL (Fig. 4). A proportion of the cultures showed the growth of very large numbers (200–1000) of clusters of 3–10 cells in the feeder layers and the feeder layer was occasionally overgrown with a diffuse " sheet " of cells. More frequently there was growth neither in the feeder layer nor in the overlay. When feeder layers were prepared using leucocytes obtained from the peripheral blood of patients with CGL whose blood counts had been restored to normal following treatment, normal colony formation was observed with normal bone marrow. Moreover, CGL leucocytes were then as good as normal leucocytes in supporting growth of CFC from CGL peripheral blood and bone marrow.

In our early fractionation experiments we routinely examined the CSF production of feeder layers consisting almost entirely (98%) of band forms and neutrophils. In numerous experiments in which neutrophils from normal donors and from

FIG. 3.—Colony forming capacity of bone marrow nucleated cells from patients with CGL related to peripheral blood leucocyte numbers.

There is a clear relationship between peripheral blood leucocyte count and bone marrow CFC numbers ($r = 0{\cdot}625$; $P < 0{\cdot}001$). The shaded area represents colony numbers in normal bone marrow. ● = untreated patients, ▲ = treated patients.

patients with untreated and treated CGL were used as feeder layers no colony formation was observed, or less than 3 colonies per plate were grown; we concluded that the mature granulocytic cells in the peripheral blood do not contribute to CSF production. Therefore, in later experiments we abandoned the use of neutrophil feeder layers. In contrast, feeder layers prepared from mononuclear cells from normal donors showed excellent CSF activity; this activity was the same whether or not we attempted to obtain a pure monocyte preparation by washing off the lymphocytes. We thus concluded that CSF production by the peripheral blood of normal donors is mainly a function of the monocytic cell component (or a fraction of it).

We considered the possibility that the poor CSF production by CGL peripheral blood leucocytes might be due to a relative scarcity of monocytes in the feeder layers. (Monocytes were present, albeit in relatively reduced numbers, in the peripheral blood of all the patients with CGL whose leucocytes were used as feeder layers.) We therefore prepared feeder layers consisting predominantly of monocytes and showed that while monocytes obtained from the peripheral blood of normal donors were capable of stimulating colony formation as well as, or better than, unfractioned leucocyte feeder layers from the same donor, feeder layers containing comparable numbers of monocytes prepared by fractionation of the peripheral blood of patients with CGL and high leucocyte counts failed to stimulate colony formation. Monocyte feeder layers, how-

FIG. 4.—CSF activity of feeder layers prepared from peripheral blood leucocytes of untreated and treated patients with CGL.

Feeder layers consisted of (1) peripheral blood leucocytes (unfractionated), (2) leucocyte fractions containing mainly monocytes, and (3) leucocyte fractions containing mainly neutrophils. Cells for the feeder layers were obtained from untreated (●) and treated (▲) patients with CGL and CSF activity was assayed by growth of CFC from normal bone marrow. The expected range of CFC numbers using feeder layers obtained from normal donors is shown as the shaded area. The horizontal bars represent means ± 1 standard deviation.

ever, prepared from the blood of patients who had received adequate treatment and whose leucocyte counts were within the normal range stimulated normal colony formation (Fig. 4).

One explanation for the lack of CSF activity in the peripheral blood of untreated patients with CGL could be that CFC already present in the feeder layers are consuming CSF locally. Because irradiation using 100–1000 rad is known to suppress colony formation without inhibiting the production of CSF, we performed experiments in which batches of feeder layers prepared from untreated patients with CGL and control donors were irradiated. The results of a representative experiment are shown (Table IV).

TABLE IV.—*CSF Activity of Feeder Layers Exposed to Differing Doses of Irradiation*

| Source of leucocytes in feeder layers | Mean colony numbers per 8 × $10^4$ normal human bone marrow cells | | | |
|---|---|---|---|---|
| | 100 rad | 500 rad | 1000 rad | No irradiation |
| CGL (untreated) | Nil | Nil | Nil | Nil |
| CGL (treated) | 25·3 (±6·3) | 33·5 (±8·5) | 28·7 (±7·4) | 33·5 (±4·7) |
| Normal subject | 20·9 (±5·9) | 25·7 (±10·1) | 22·7 (±5·2) | 27·5 (±10·0) |

Figures are means from one experiment ± 1 standard deviation.

We concluded that if available CSF was being consumed by CFC present in the feeder layers, irradiation should block this consumption and so allow its release to support growth in the overlay. This was not observed.

### DISCUSSION

Our experiments were designed to test two properties of the haemopoietic cells of patients with CGL. First we examined the colony forming capacity in agar of peripheral blood and bone marrow cells obtained from patients before and after treatment; secondly, we examined the capacity of peripheral blood leucocytes from such patients to support the growth in agar culture of normal human bone marrow cells—that is, their CSF activity. Our experiments on the colony forming capacity of cells from patients with CGL have confirmed those of other workers, and we have shown that peripheral blood and bone marrow CFC numbers are approximately proportional to the peripheral blood leucocyte count. In contrast, our experiments on the CSF activity of cells from patients with CGL have yielded results that conflict with previous reports; we have found that the leucocytes of patients with untreated CGL lack CSF activity while their CSF activity is in the normal range when the peripheral blood leucocyte count has been restored to normal by treatment. We have also shown that the CSF activity of the leucocytes of the treated patients is associated mainly with the monocytes, while the monocytes of untreated patients are inactive.

### *Colony forming capacity of leucocytes in CGL*

The observation that CFC appear in the peripheral blood in greatly increased numbers in the untreated patient with CGL is not new (Paran *et al.*, 1970; Moore *et al.*, 1973*b*). It has been estimated that the normal individual has about 10 CFC per $10^6$ nucleated cells in the peripheral blood (Chervenick and Boggs, 1971) but in untreated CGL this number may rise to 1000–2000 and was found to average 1350 in this study. In addition, the number of cluster forming cells in the peripheral blood, presumably representing differentiated granulocytic cells, may be extremely high (Moore *et al.*, 1973*b*) but such clusters are readily distinguished from normal colonies and have been ignored for the purpose of this study. The relative increase in CFC in the bone marrow of untreated patients appears to be much less and only of the order of two- or three-fold; the absolute increase is, of course, much greater as the total myeloid mass of untreated patients is characteristically greatly enlarged. When the total leucocyte count has been restored to normal and the blast cells have disappeared from the peripheral blood, we find that CFC are greatly reduced or absent in the peripheral blood and appear to be of approximately normal number in the marrow. This agrees well with the observations of other authors (Moore *et al.*, 1973*b*).

When we expressed CFC numbers as a proportion of nucleated peripheral blood or bone marrow cells plated, we observed a correlation between CFC numbers on the one hand and the total peripheral blood leucocyte count of the CGL donor on the other. Inasmuch as the total leucocyte count gives some indication of the increased total granulocyte mass of the patient and thus of the extent of the disease, this general correlation is perhaps not surprising. What did surprise us was the apparent log–log nature of the relationship: for any given increase in leucocyte count there was a proportionately greater increase in circulating CFC numbers. We doubt if this is explained by a non-linear relationship between observed colony numbers and CFC plated since the effect of plating larger numbers of CFC in each dish is more often to reduce proportionately than to increase numbers. More likely the observation is real and advancing disease leads to the circulation of proportionately greater numbers of CFC. We also noted a correlation between the peripheral blood count and the number of CFC present in the bone marrow.

We might also have expected to have shown a general relationship between the number of peripheral blood blast cells plated and the number of colonies observed in a particular experiment. No such correlation was observed. This could be due to the inherent inaccuracy of calculating the absolute number of blast cells plated from a differential count involving a rather low percentage of such cells. Alternatively, it may mean that there is no exact relationship between the numbers of CFC and of blast cells present in a given volume of blood. In this context, it is of interest to note that the cell tentatively identified as the haemopoietic stem cell in certain primates (Dicke *et al.*, 1973) bears no morphological resemblance to the myeloid blast cell of CGL.

*Colony stimulating activity of leucocytes in CGL*

Leucocytes obtained from the peripheral blood of normal persons are in most cases an excellent source of CSF and this appears to originate from the monocytic cell component (Golde and Cline, 1972; Golde, Finley and Cline, 1972; Chervenick and LoBuglio, 1972). We have now shown that the peripheral blood leucocytes of untreated patients with CGL are a very poor source of CSF, and this observation conflicts with the data of Moore who reported uniformly good feeder layer activity from patients with CGL (Moore, Williams and Metcalf, 1973*a*). The discrepant findings could be explained by the fact that not all Moore's patients were untreated or by the fact that the CFC present in mouse bone marrow, used in many of Moore's experiments, may differ in CSF requirements from human CFC. We found that peripheral blood leucocyte feeder layers prepared from patients whose peripheral blood counts had been restored to normal by treatment had normal CSF activity. This was a uniform finding in our group of treated patients, which included some whose peripheral blood had been shown to be devoid of CSF activity before treatment.

The finding that the peripheral blood of untreated patients with CGL does not provide CSF could be explained in a number of ways. In the first place it is possible that CSF is produced but consumed locally by CFC and cluster forming cells present in the feeder layer. We believe that the finding that irradiated CGL feeder layers, in which all cellular proliferation has been suppressed, still fail to produce CSF makes this explanation unlikely. Secondly, our findings would be adequately explained by high inhibitor levels in the sera of untreated patients with CGL but in fact very little autologous plasma can be present in the feeder layers after the leucocytes have been washed 2 or 3 times. Moreover, patients with CGL have for the most part normal serum inhibitor levels (Metcalf, 1973). Thirdly, it is conceivable that CGL leucocyte feeder layers produce little CSF because of a relative scarcity of monocytes; some monocytes were, however, present in all our leucocyte feeder layers. In addition, the number of monocytes obtained from donors with CGL for use as purified monocyte feeder layers was approximately comparable with that obtained from normal donors; the former still failed to show CSF activity. Purified monocyte feeder layers obtained from patients after treatment had normal CSF activity. We are led to the conclusion that the monocyte present in the peripheral blood of untreated patients fails to produce CSF either because its normal CSF production has been " switched off " or because it is intrinsically defective.

This last conclusion may be expanded into two different hypotheses which are presumably mutually exclusive. One explanation hinges on the possible role of CSF as a physiological regulator of peripheral blood granulocyte numbers. In other words, it is possible that the large number of granulocytes in the peripheral blood in CGL leads to suppression of CSF production by functionally normal monocytes. (Presumably such suppression is mediated in a manner distinct from the inhibitors that have been identified in the sera of normal people.) When tested *in vitro*, these suppressed monocytes still fail to produce CSF even though they are no longer directly exposed to the large granulocyte population. When treatment reduces the total granulocyte mass and the peripheral blood granulocyte numbers, the negative feedback to the monocyte is removed and CSF is again produced. On this hypothesis the functionally normal monocyte (*i.e.* a cell sensitive to suppression by negative feedback from granulocytes and also capable of producing CSF) might be a member of the CGL clone of myeloid cells; however, it is not yet known whether monocytes in CGL carry the $Ph^1$ marker chromosome. The alternative explanation implies that there are two distinct monocyte populations, one intrinsically defective in CSF production that predominates in the peripheral blood before treatment, and the other, indistinguishable from the normal monocyte population, which proliferates after treatment. The data necessary to distinguish these two possibilities are not yet available.

We are most grateful to the other members of the M.R.C. Leukaemia Unit and to Dr George Marsh (North Middlesex Hospital) and Dr Paul Roberts (West Middlesex Hospital) whose patients are included in this study. We thank Dr D. A. G. Galton for invaluable criticism of the manuscript. Secretarial help was provided by Mrs Day Haysome and Miss Margot Kuhne.

### REFERENCES

BÖYUM, A. (1968) Isolation of Mononuclear Cells and Granulocytes from Human Blood. *Scand. J. clin. lab. Invest.*, Suppl. 97, **21**, 77.

BROWN, C. H. & CARBONE, P. P. (1971) *In vitro* Growth of Normal and Leukemic Human Bone marrow. *J. natn. Cancer Inst.*, **46**, 989.

CHERVENICK, P. A. & BOGGS, D. R. (1971) *In vitro* Growth of Granulocytic and Mononuclear Colonies from Blood of Normal Individuals. *Blood*, **37**, 131.

CHERVENICK, P. A., ELLIS, L. D., PAN, S. F. & LAWSON, A. L. (1971) Human Leukemic Cells: *in vitro* Growth of Colonies Containing the Philadelphia ($Ph^1$) Chromosome. *Science, N.Y.*, **174**, 1134.

CHERVENICK, P. A. & LOBUGLIO, A. F. (1972) Human Blood Monocytes: Stimulators of Granulocyte and Mononuclear Colony Formation *in vitro*. *Science, N.Y.*, **178**, 164.

DICKE, K. A., VAN NOORD, M. J., MAAT, B., SCHAEFER, U. W. & VAN BEKKUM, D. W. (1973) Attempts at Morphological Identification of the Haemopoietic Stem Cell in Primates and Rodents. In *Haemopoietic Stem Cells*. Ed. G. E. W. Wolstenholme. (Ciba Foundation Symposium 13, new series). Amsterdam: North Holland.

GOLDE, D. W. & CLINE, M. J. (1972) Identification of the Colony-stimulating Cell in Human Peripheral Blood. *J. clin. Invest.*, **51**, 2981.

GOLDE, D. W., FINLEY, T. N. & CLINE, M. J. (1972) Production of Colony-stimulating Factor by Human Macrophages. *Lancet*, ii, 1397.

GREENBERG, P. L., NICHOLS, W. C. & SCHRIER, S. L. (1971) Granulopoiesis in Acute Myeloid Leukemia and Preleukemia. *New Engl. J. Med.*, **284**, 1225.

METCALF, D. (1973) Human Leukaemia: Recent Tissue Culture Studies on the Nature of Myeloid Leukaemia. *Br. J. Cancer*, **27**, 191.

MOORE, M. A. S., WILLIAMS, N. & METCALF, D. (1973a) *In vitro* Colony Formation by Normal and Leukemic Human Hematopoietic Cells: Interaction between Colony-forming and Colony-stimulating Cells. *J. natn. Cancer Inst.*, **50**, 591.

MOORE, M. A. S., WILLIAMS, N. & METCALF, D. (1973b) *In vitro* Colony Formation by Normal and Leukemic Human Hematopoietic Cells: Characterization of the Colony-forming Cells. *J. natn. Cancer Inst.*, **50**, 603.

PARAN, M., SACHS, L., BARAK, Y. & RESNITZKY, P. (1970) *In vitro* Induction of Granulocyte Differentiation in Hematopoietic Cells from Leukemic and Non-leukemic Patients. *Proc. natn. Acad. Sci. U.S.A.*, **67**, 1542.

PIKE, B. L. & ROBINSON, W. A. (1970) Human Bone Marrow Colony Growth in Agar-gel. *J. cell. comp. Physiol.*, **76**, 77.

ROBINSON, W. A. & PIKE, B. L. (1970) Colony Growth of Human Bone Marrow Cells *in vitro*. In *Hemopoietic Cellular Proliferation*. Ed. F. Stohlman Jr. New York: Grune & Stratton. p. 249.

SHADDUCK, R. K. & NANKIN, H. R. (1971) Cellular Origin of Granulocyte Colonies in Chronic Myeloid Leukaemia. *Lancet*, ii, 1097.